# Tuning into flavor: predicting coffee sensory attributes from EEG with boosted-tree regression models

**DOI:** 10.3389/fnhum.2025.1661214

**Published:** 2025-10-09

**Authors:** Marco Bilucaglia, Mara Bellati, Alessandro Fici, Vincenzo Russo, Margherita Zito

**Affiliations:** ^1^Behavior and Brain Laboratory IULM – Neuromarketing Research Center, Università IULM, Milan, Italy; ^2^Department of Business, Law, Economics and Consumer Behaviour “Carlo A. Ricciardi, ” Università IULM, Milan, Italy; ^3^Department of Philosophy and Cultural Heritage, Ca' Foscari University, Venice, Italy

**Keywords:** coffee flavor prediction, electroencephalography (EEG), machine learning (ML), ensemble learning, boosted-tree regression, Descriptive Sensory Analysis (DSA)

## Abstract

Flavor, a multimodal perception based on taste, smell, and tactile cues, plays a significant role in consumer preferences and purchase intentions toward coffee. In this exploratory study, we assessed the potential of electroencephalography (EEG) and machine learning (ML) techniques to predict coffee sensory attributes. We extracted spectral and temporal features from a professional panel while tasting coffee samples and basic water solutions. We trained multiple Least-Squares Boosted Trees (LSBoost) and optimized their hyperparameters through a 100-step Bayesian approach based on a Leave-One-Subject-Out (LOSO) scheme. The models achieved, overall, high predictive accuracy (MAE < 0.75 on a 0 − 10 scale) and medium-to-large robustness (Cohen's*d*>0.6) with respect to mean and lasso benchmark regressors. Feature importance analysis revealed that spectral powers and Hjorth's parameters within parietal, central, and frontal regions were the most predictive. Our findings endorse the use of EEG-based ML models as an alternative to traditional flavor evaluation methods, such as Descriptive Sensory Analysis (DSA).

## 1 Introduction

Coffee stands as the major component of the global hot drink market, with a worldwide production exceeding 176 million bags and consumption reaching 7 billion kilograms (Kim Y. et al., 2025). Among over 60 coffee plant species, only 10 are extensively cultivated, with *Coffea arabica* (arabica), *Coffea canephora* (robusta), and *Coffea liberica* being the most prevalent (Feria-Morales, 2002). Arabica and robusta make up 99% of global production (Jayakumar et al., 2017) and commercial coffee typically results from blending their beans in varying proportions (Seninde and Chambers, 2020).

Consumer preference and purchasing intentions for coffee are mainly driven by subjective factors, such as taste, aroma, and body (Li et al., 2019). These elements belong to the broader concept of flavor, a multimodal experience in which gustatory, olfactory, trigeminal, and somatosensory inputs are individually processed before being integrated (Small, 2012). Gustatory signals ascend via the nucleus of the solitary tract (NST) and ventroposteromedial nucleus (VPM) to the primary taste cortex in the rostral insula and adjoining frontal operculum, where the identity and intensity of basic tastes, as well as oral texture and temperature, are encoded. Retronasal olfactory inputs, initially processed in the piriform cortex, converge with gustatory signals in the orbitofrontal cortex (OFC), which also integrates oral somatosensory and trigeminal inputs such as viscosity, temperature, irritation, and astringency (Rolls, 2005). Within the OFC, convergent inputs give rise to multimodal flavor representations, in which sensory modalities are integrated and assigned hedonic value. At the same time, projections to the amygdala and anterior cingulate cortex (ACC) further embed these representations within affective and motivational systems (Small, 2012).

Flavor assessment often relies on Descriptive Sensory Analysis (DSA), wherein expert panels assign numerical scores to standardized sensory attributes (Yang and Lee, 2019). Various coffee attributes have been suggested (Spencer et al., 2016). However, only bitter, sour, sweet, and astringent (i.e., mouth-drying sensation) have established reference solutions (Batali et al., 2022).

Based on self-reports, DSA can be confounded by physiological and psychological biases. Physiological phenomena include sensory adaptation and multimodal enhancement/suppression. Psychological phenomena include expectation, stimulus/proximity/logical errors, habituation, halo effect, presentation order, mutual suggestion, and central/extreme rating tendency (Stone et al., 2021; Civille et al., 2024). To mitigate these risks, international standards (e.g., ISO 13299, ISO 11132, and ISO 8586) and the scientific literature recommend extraneous cues blinding, randomized or Williams-balanced presentations, adequate rests/rinses alternation, and ongoing panel performance monitoring (Sipos et al., 2021). Additionally, direct techniques based on bioelectrical measures have recently been advocated (Torrico et al., 2023; Rodrigues et al., 2024).

Previous studies have explored the use of electroencephalography (EEG) for flavor assessment. Global field power and scalp topographies (Crouzet et al., 2015), as well as phase in the delta band (Wallroth et al., 2018) and spectral powers in alpha and theta bands (Yang et al., 2023) have emerged as candidate neurometrics. Similar results, involving alpha, beta, and theta powers, have been observed in coffee tasting tasks (Hsu and Chen, 2021; Tonacci et al., 2024). However, their correlational—rather than causal—nature poses a risk of reverse inference problems (Poldrack, 2006). Pattern-decoding methods based on Machine Learning (ML) models have been suggested to mitigate this issue (Nathan and Del Pinal, 2017). Furthermore, being free of rigid theoretical assumptions, ML methods could also be helpful in revealing latent structures in the data, providing new theoretical insights and hypotheses (Verzelli et al., 2024).

Research on flavor prediction with ML and EEG data is still limited (see the following Section 2 for details). Most of the studies employed basic water solutions as eliciting stimuli, and the few examining coffee focused on other target variables than taste. Moreover, nearly all existing models were classifiers to discriminate among basic tastes (e.g., sour, sweet, bitter, salty, umami, and neutral) instead of predicting the intensity level of sensory attributes. Therefore, such models are ill-suited to replace or even complement traditional DSA.

To address these limitations, we performed an exploratory study recording the EEG data from expert coffee tasters while they tasted both reference solutions and coffee samples. We trained multiple tree-based ensemble regressors to predict the intensity level of bitter, sweet, acid and astringent, achieving high performances and robustness against benchmark models. We interpreted the fitted models, identifying as most informative, spectral and temporal features within parietal, central and frontal regions.

## 2 Related work

As previously mentioned, most of the past studies on taste prediction using EEG data and ML methods trained classifiers to discriminate among basic water solutions. De et al. (2023) fed temporal (maximum/minimum values, mean, kurtosis and skewness) and spectral [Power Spectral Densities (PSDs) in theta, delta, alpha and beta bands] features into a Long Short-Term Memory Recurrent Neural Network (LSTM-RNN) to discriminate sour, sweet, bitter, salty, umami, and neutral solutions from 46 subjects. They obtained an accuracy of 97.16%. Xia et al. (2024) employed a Convolutional Neural Network (CNN) with spatiotemporally augmented raw EEG data to identify sour, sweet, bitter, and salty solutions from 20 subjects. They reached 99.5% of accuracy. Li et al. (2025) trained a Support Vector Machine (SVM) with spectral features (wavelet decompositions in α and θ bands) to classify sour, sweet, bitter, salty, and umami solutions from 22 subjects. They reported a maximum accuracy of 76.13%. Vo et al. (2023) trained a feed-forward Neural Network (NN) using spectral features (powers in delta, theta, alpha, beta, and gamma bands) to discriminate between salty and sour solutions from 15 people. The accuracy was 84.36%.

Only one study moved from discrimination to intensity level prediction. Zhao et al. (2022) contrasted linear, tree, and ensemble regressors, trained with temporal and information-related features (energy, absolute mean value, and wavelet entropy), to predict the intensity level of sour, sweet, bitter, salty, and umami from 10 subjects. The best model, Extreme Gradient Boosting (XGBoost), achieved a goodness-of-fit (measured through the *R*^2^ coefficient), ranging from −0.22 to 0.18.

Two studies focused on other-than-basic water solutions. González-España et al. (2023) aimed to discriminate wine vs. water and wine vs. wine tasting tasks of 10 participants through an SVM with temporal and spatial features (global field powers and channel averages). They reported accuracies greater than the chance level of 70% for both predictions. Naser and Aydemir (2024) trained a k-Nearest Neighbors (kNN) and a Random Forest (RF) with temporal and spectral features (mean value of the Hilbert-transformed EEG signal and level-2 wavelet coefficients) to discriminate four food substances (oils of Orange, Mint, Thyme, and Clove) from 10 subjects. The highest accuracy, obtained with the kNN, was 87.5%.

Coffee was selected as an eliciting stimulus in two studies. However, as previously mentioned, the target belonged to other aspects than taste. Maram et al. (2023) trained a CNN with raw EEG data to classify the preference of 3 coffee brands from 12 participants, obtaining an accuracy of 83.43%. Xu et al. (2021) compared several Bayesian Regression (BR) models, trained with spectral features (powers in theta, alpha, beta, and gamma bands), to predict the emotional responses to tasting tasks from 32 subjects. The best model achieved a goodness-of-fit [measured through the Watanabe-Akaike Information Criterion (WAIC)] of 963.55.

## 3 Materials and methods

### 3.1 Study population

A total of 15 subjects (9 females) in the age range 24–59 years (*M* = 40.13, *SD* = 13.80) took part in the experiment. They were recruited as professional coffee tasters with proficiency in DSA and grouped as trained (T, less than 3 years of experience) or experts (E, more than 3 years of experience). Despite the sample size being below the average when compared to the surveyed past studies (i.e. 19.67 ± 12.31, range: 10 − 46), it was still in line with DSA studies that typically consist of 5–15 experts (Gacula and Rutenbeck, 2006).

The participants resulted group- and gender-balanced in terms of mean age [*t*(13) = 0.818, *p* = 0.428 and *t*(13) = 1.034, *p* = 0.320, respectively]. However, the groups resulted in an unbalanced in terms of gender proportion [E: 2 females, T: 7 females, χ^2^(1) = 5.402, *p* = 0.020].

A sensitivity analysis performed with G*Power (Faul et al., 2007) considering a within-between design with 2 groups, 8 measures, and standard parameters (α = 0.05, 1−β = 0.95, ϵ = 1, ρ = 0.5) confirmed a minimum detectable effect size of *f* = 0.302, interpreted as medium-to-large (Cohen, 1992).

The study was approved by the Ethical Committee of Universitá IULM (approval number: 0067814). All the procedures adhered to the guidelines of the Helsinki Declaration, and informed consent was secured from each participant.

### 3.2 Instrumentation

The EEG was acquired using the NVX-52 device (Medical Computer System Ltd.) from 38 Ag/AgCl scalp electrodes, 2 Ag/AgCl ear clips (A1 and A2), and 1 adhesive Ag/AgCl patch placed on the left mastoids (M1). The electrode positioning, detailed in Bilucaglia et al. (2024), followed the 10-10 system (Nuwer, 2018), and the montage was monopolar, reference-free, and grounded to M1. Neorec software (Medical Computer System Ltd) was used to record the data at a sample frequency of 2*kHz* and a resolution of 24*bits*.

The iMotions software (iMotions A/S) was used to deliver the experiment instructions and collect the sensory evaluations.

Data synchronization was ensured by a transistor-to-transistor (TTL) pulse, sent by iMotions at the beginning of the experiment and fed into the NVX-52 by means of the ESB synchronization box (Bilucaglia et al., 2020).

All computations were carried out on a workstation equipped with an AMD Ryzen™ Threadripper PRO 5975WX CPU (32 cores, 64 threads, 3.6 GHz base clock) and 256 GB of DDR4-3,200 MHz ECC. No GPU acceleration was used. Code was executed in MATLAB R2024b (The Mathworks, Inc.) with Statistics and Machine Learning Toolbox 12.3.

### 3.3 Experimental protocol

The experiment consisted of a starting 60*s* eye-closed baseline (BSL) and two experimental phases, namely benchmark (Be) and coffee (Co).

The Be phase involved 4 tasting trials with solutions of sucrose (20*g*/*l*), caffeine (0.6*g*/*l*), citric acid (0.6*g*/*l*), and potassium aluminum sulfate (1*g*/*l*), to elicit sweet, bitter acid, and astringent flavors respectively (Anbarasan et al., 2022). Micro-filtered mineral water was used as diluent. According to Rousmans et al. (2000), the exact concentration of the solutions was determined from a previous pilot test.

According to Abubakar et al. (2020), the Co phase involved four tasting trials with coffees at various arabica/robusta ratios (100:0, 80:20, 85:15, and 70:30).

The phase order was fixed (i.e., first Be and then Co), and the tasting trials were randomized within each phase.

The administration of liquids was masked. The Be solutions were served at room temperature, while the Co at approximately 60°C. According to Di Flumeri et al. (2017), participants were instructed to rinse the palate with a glass of water before any tasting trial (WR task) and to keep the liquid (solution or coffee) in the mouth for 10*s* (TL task) before swallowing. At the end of each tasting trial, subjective ratings for bitter, astringent, sweet, and acid attributes were collected on a 0 − 10 scale.

**Figure 1:**
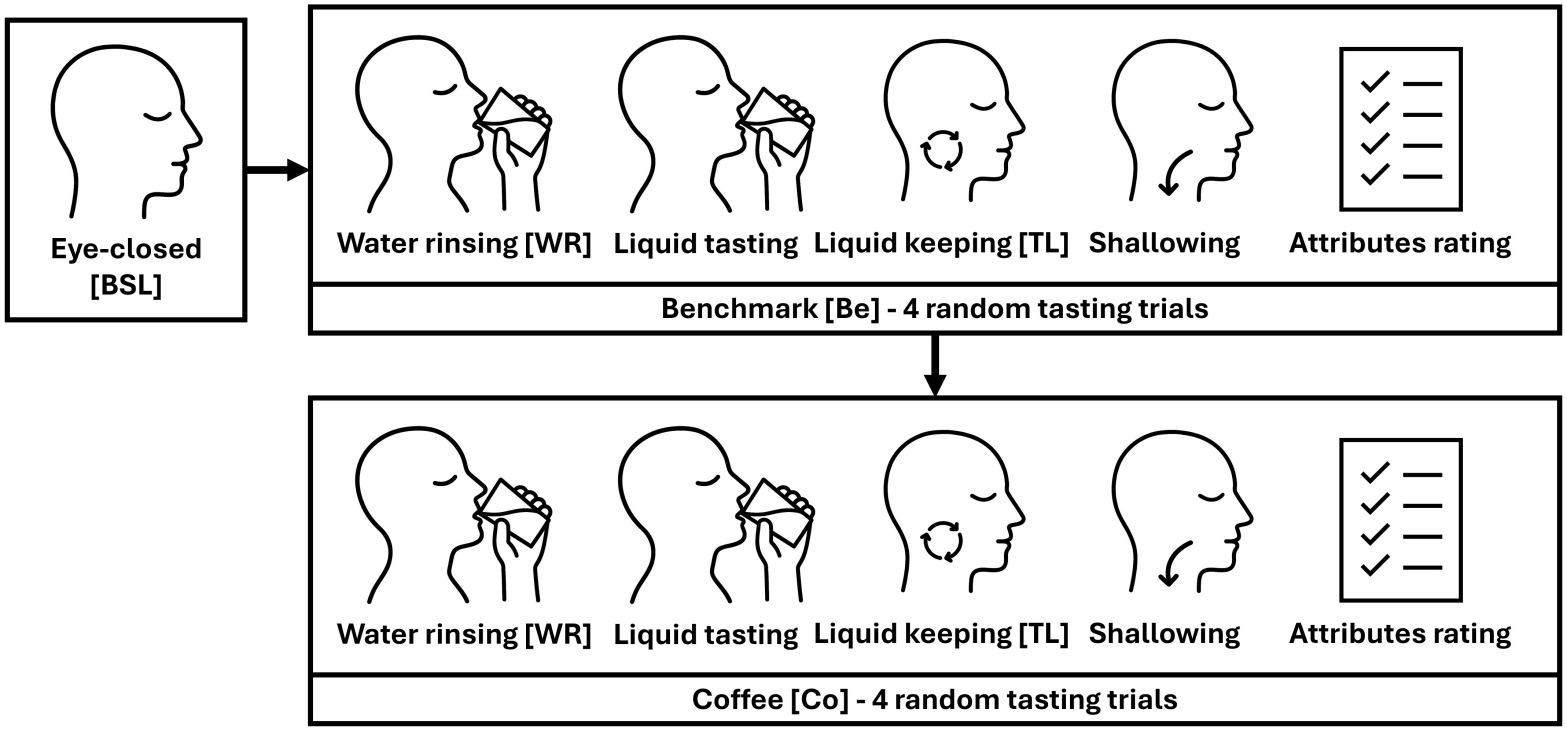
Schematic representation of the experimental protocol.

The following Figure 1 summarizes the experimental protocol.

### 3.4 Data processing

Data processing was performed using the EEGLab toolbox (Delorme and Makeig, 2004). The EEG was resampled at 512*Hz* and filtered in the 0.1 − 40*Hz* band (IV zero-phase Butterworth filter). Power line interference (50 and 100*Hz*) was reduced through the CleanLine, a multi-taper-based regression technique (Bokil et al., 2010), while non-stationary artifacts were corrected using the Artifact Subspace Reconstruction (ASR) method (Chang et al., 2020) with standard cutoff parameter (κ = 20). ASR represents the gold standard for handling high-amplitude artifacts, such as those related to locomotor tasks in real-world and Mobile Brain Imaging (MoBI) contexts (Kim H. et al., 2025). Independent component analysis (ICA) decomposition was carried out using the second-order blind identification (SOBI) algorithm (Urigüen and Garcia-Zapirain, 2015) on a resampled (100*Hz*) and heavily filtered (1 − 30*Hz*, IV order zero-phase Butterworth filter) copy of the data. According to Bilucaglia et al. (2024), the resulting weight matrix was multiplied by the original data to obtain the independent components (ICs). The ICLabel classifier (Pion-Tonachini et al., 2019) was used to detect artifactual ICs as those with not-brain probability Pr{!brain}≥0.9. On average, 3.8 ± 1.373 (min = 2, max = 7) artifactual ICs over 38 were identified and removed. Finally, a re-reference to the approximately zero ideal potential was performed through the Representational State Transfer (REST) algorithm (Yao, 2001).

**Figure 2:**
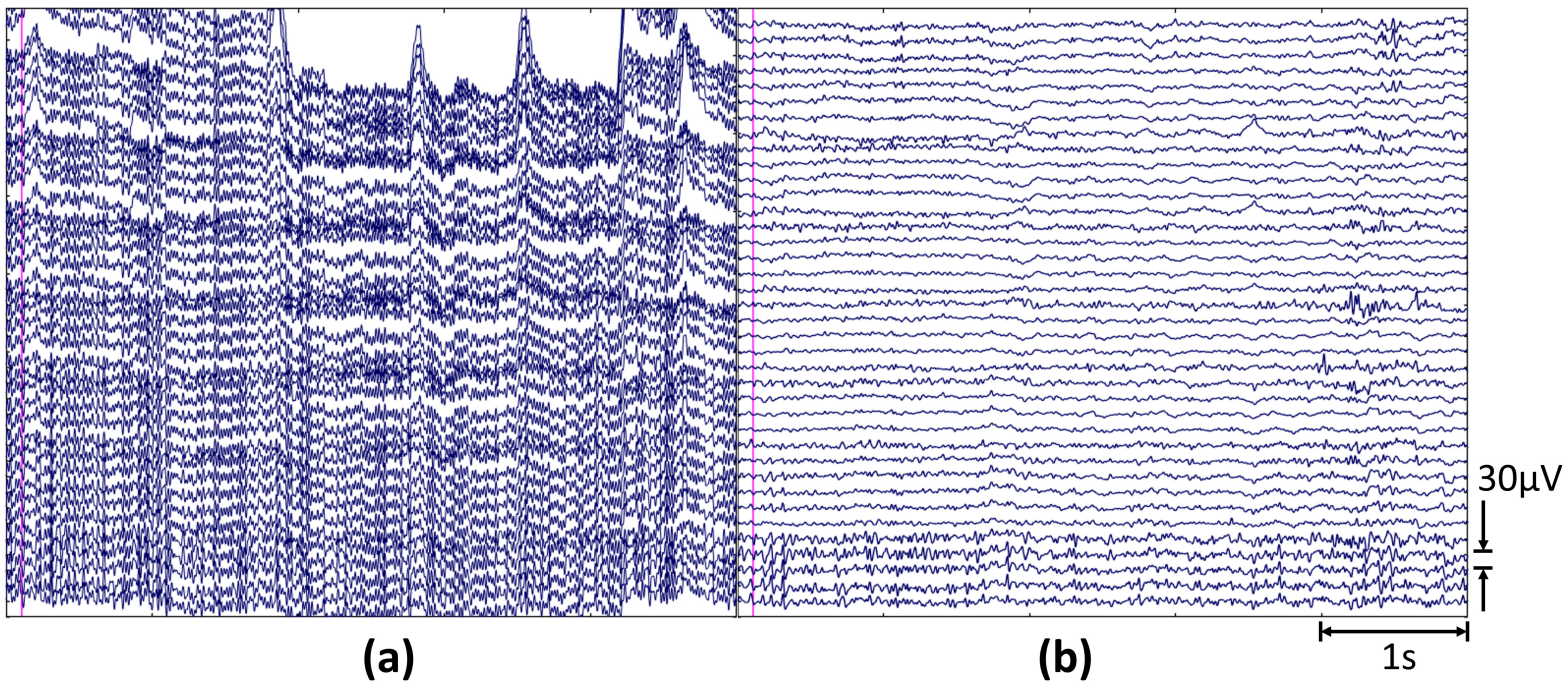
Representative 5*s*−long segments showing the **(a)** raw and **(b)** pre-processed EEG signal. The data refer to the TL task of As solution, with onset marked by the pink vertical line.

The cleaned EEG was offline aligned to the starting TTL pulse and epoched according to the experimental phases (i.e., EYC and TL tasks of Be and Co). The following Figure 2 shows a representative segment of raw and pre-processed EEG data.

For each subject, the Individual Alpha Frequency (IAF) was computed as the center of gravity of the Power Spectral Density (PSD) in the extended (7.5 − 12.5*Hz*) alpha band (Klimesch, 1999). As PSD, the average occipital Welch's PSD (1*s*−long Hamming window at 50% of overlapping) estimated in the BSL epoch was considered (Bilucaglia et al., 2019). The IAF served to define the following subject-specific δ, θ, α, β, and γ EEG bands (Borghini et al., 2019):


(1) $$\begin{array}{l} \delta =\left[0,\text{IAF}-6\right]\\ \theta =\left[\text{IAF}-6,\text{IAF}-2\right]\\ \alpha =\left[\text{IAF}-2,\text{IAF}+2\right]\\ \beta =\left[\text{IAF}+2,\text{IAF}+16\right]\\ \gamma =\left[\text{IAF}+16,\text{IAF}+25\right] \end{array}$$

### 3.5 Feature extraction

Features were extracted exclusively from WR and TL epochs, thereby excluding non-task-related activity that could also have been potentially contaminated by residual muscle artifacts. For each channel *C* and band *B* = {δ, θ, α, β, γ}, normalized spectral powers *p*_*C, B*_ were computed as (Bilucaglia et al., 2022):


(2) $$\begin{array}{l} p_{C,B}=\frac{\int_{B}x_{C}\left(f\right)df}{\int_{-\infty }^{+\infty }x_{C}\left(f\right)df}, \end{array}$$

where *x*_*C*_(*f*) is the Welch's PSD (1*s*−long Hamming window with 50% of overlapping).

Additionally, the following activity (*A*_*C*_), mobility (*M*_*C*_), and complexity (*C*_*C*_) temporal parameters were computed as (Hjorth, 1970):


(3) $$\begin{array}{l} A_{C}\left\{x_{C}\left(t\right)\right\}=\sigma \left\{x_{C}\left(t\right)\right\}\\ M_{C}\left\{x_{C}\left(t\right)\right\}=\sqrt{\frac{A_{C}\left\{\frac{dx_{c}\left(t\right)}{dt}\right\}}{A_{C}\left\{x_{C}\left(t\right)\right\}}},\\ C_{C}\left\{x_{C}\left(t\right)\right\}=\frac{M_{C}\left\{\frac{dx_{c}\left(t\right)}{dt}\right\}}{M_{C}\left\{x_{C}\left(t\right)\right\}} \end{array}$$

where σ{·} is the temporal variance operator.

Expertise level (group) and age were also considered, since their impact on flavor evaluation has been previously reported (Croijmans and Majid, 2016; Mojet, 2003).

The feature vectors (306 − long) were finally obtained by concatenating spectral (38 × 5 = 190) and temporal (38 × 3 = 114) vectors, as well as age and group (categorical: T, E) scalars. For each tasting trial *T* and each phase *P*, the TL vectors ***x***_*T, P*_ were normalized with respect to the WL vectors ***y***_*T, P*_ as:


(4) $$\begin{array}{l} x_{T,P}^{\prime }=\left(x_{T,P}-y_{T,P}\right)⊘y_{T,P} \end{array}$$

where ⊘ represents the Hadamard (i.e., element-wise) division operator.

Three datasets corresponding to the Be and Co phases (60 × 306 each) as well as the BeCo (120 × 360) consisting of the normalized TL vectors $x_{T,P}^{\prime }$ were finally built. The target variables *y*, consisting of the attribute ratings, were transformed as log(1+*y*), following general recommendations for ratio scales (Keene, 1995).

### 3.6 Model training and evaluation

The selected model was LSBoost, a least-squares variant of Boosted Trees (Friedman, 2001). It was chosen for the enhanced predictive performance, as a non-linear ensemble method, and because it incorporates feature selection within the weak learners (Decision Trees, DTs). Regressors belonging to the boosted trees family have been previously shown to outperform in EEG prediction tasks (Hussain et al., 2019; He et al., 2022; Isabona et al., 2022).

LSBoost's tunable hyperparameters included the number of learners (*n*) and the learning rate (ρ), while DTs' ones included the leaf size (*l*_*s*_) and the maximum number of splits (*n*_*s*_). Since the dataset pre-processing is known to impact the performance of the EEG-based prediction models (Apicella et al., 2023; Tryon et al., 2025), different standardization techniques (*S*) were also considered. They included the subject-wise z-score and min-max normalisations, as well as a non-linear transformation based on the median value (Arevalillo-Herráez et al., 2019) and the lack of standardization (none).

The best hyperparameters $\left(n^{*},\rho^{*},l_{s}^{*},n_{s}^{*},S^{*}\right)$ were obtained through a Bayesian optimization (Snoek et al., 2012), considering the cross-validated Mean Absolute Error (MAE) as objective function L. This solved the following Combined Algorithm Selection and Hyperparameter (CASH) problem (Kotthoff et al., 2017):


(5) $$\begin{array}{l} \left(n^{*},\rho^{*},l_{s}^{*},n_{s}^{*},S^{*}\right)=\underset{n,\rho ,l_{s},n_{s},S}{\mathrm{argmin}}L\left(n,\rho ,l_{s},n_{s},S\right), \end{array}$$

where *n*∈[1 − 500], ρ∈[0.01, 1], *l*_*s*_, *n*_*s*_∈[1 − 100] and *S*∈{z-score, minmax, median, none}.

The maximum number of evaluations was set to 100 and the seed was fixed at rng(1), to ensure replicability.

The cross-validation followed a Leave-One-Subject-Out (LOSO) (Fazli et al., 2009) scheme to address the subject-wise data dependence.

As baselines for a robust model evaluation, a regressor constantly predicting the training set's mean target (Me) and a lasso regressor (LR) (Tibshirani, 1996) were additionally fitted following the same LOSO approach. The lasso's penalisation term λ was set at (Bühlmann and Van De Geer, 2011, p. 14):


(6) $$\begin{array}{l} \lambda =\sqrt{2\log\left(p\right)/n}, \end{array}$$

where *n* = 56 and *p* = 306 are the dimensions of the training set.

The cross-validated MAEs of LSBoost vs. Me and LBoost vs. LR were compared by means of the following Cohen's d-scores (Goulet-Pelletier and Cousineau, 2018):


(7) $$\begin{array}{l} d_{Me,LR}=\frac{m_{Me,LR}-m_{LSBoost}}{{\bar{s}}_{Me,LR}} \end{array}$$

where ${\bar{s}}_{Me,LR}=\sqrt{\left(s_{Me,LR}^{2}+s_{LSboost}^{2}\right)/2}$, *m*_(·)_ and *s*_(·)_ are the mean and standard deviation of the cross-validated MAEs, respectively. Cutoffs for small, medium, and large differences are placed at 0.2, 0.5, and 0.8, respectively (Cohen, 1992).

The significance at α = 0.05 level of each *d* coefficient was assessed from its 95% Confidence Intervals, estimated from a non-central *t* distribution (Goulet-Pelletier and Cousineau, 2018).

To assess the predictive power of the features, models showing significant *d*_*Me, LR*_ coefficients were trained on the complete datasets using optimal hyperparameters. Then, LSBoost's feature importance scores were extracted, normalized to the total importance score, and then summed across the channels. According to Bilucaglia et al. (2019), a topographic map showing the feature importance distribution was then obtained by averaging the scores across datasets, targets, and models.

## 4 Results

Four models reported significant improvements from the benchmarks, with an overall MAE of 0.537 ± 0.073 (anti-log:0.714 ± 0.124) and *d* scores of *d*_*Me*_ = 0.858 ± 0.341, *d*_*LR*_ = 0.897 ± 0.326. The targets were Bi (trained on Be), Sw (on Be), Ac (on Co), and As (on BeCo).

The Ac prediction obtained the best MAE (Co: 0.459 ± 0.178), while Sw the worst one (Be: 0.600 ± 0.282). The highest robustness against benchmark regressors was achieved by Bi trained on Be (*d*_*Me*_ = 1.344, *d*_*LR*_ = 1.372), while the lowest one was observed for Sw trained on Be (*d*_*Me*_ = 0.651, *d*_*LR*_ = 0.660).

The following Table 1 reports the best hyperparameters and the training time (in seconds) of the significant models, split for dataset and target. The following Table 2 summarizes the performances (cross-validated MAEs and Cohen's *d* coefficients) of the significant models, split for datasets and targets.

**Table 1:** Significant models with best hyper-parameters and training time (in seconds) split for dataset and target.

** D **	** T **	***n***	**ρ**	***l*_*s*_**	***n*_*s*_**	***S***	**t**
Be	Bi	20	0.078	4	27	Z-score	38.115
Be	Sw	16	0.657	4	4	Median	41.324
Co	Ac	4	0.531	10	79	Median	35.779
BeCo	As	38	0.075	13	96	Min-max	37.162

D, dataset; T, target; *n*, number of learners; ρ, learning rate; *l*_*s*_, leaf size; *n*_*s*_, number of splits; *S*, standardization method; *t*, training time [s].

**Table 2:** Performances of the significant models split for the dataset and target.

D	T	**LSBoost**		**Me**		**LR**		***d*_*Me*_**	***d*_*LR*_**
		**M**	**SD**	**M**	**SD**	**M**	**SD**		
Be	Bi	0.490	0.176	0.694	0.127	0.692	0.118	1.344	1.372
Be	Sw	0.600	0.282	0.743	0.158	0.774	0.247	0.651	0.660
Co	Ac	0.459	0.178	0.604	0.165	1.008	1.125	0.844	0.841
BeCo	As	0.598	0.132	0.681	0.146	1.058	1.153	0.595	0.717

D, dataset; T, target; LSBoost, Least-Square Boosted Tree; Me, mean regressor; LR, lasso regressor; M, mean; SD, standard deviation; *d*, Cohen's d.

Both spectral and temporal features contributed to the predictions, but their importance scores varied substantially across datasets and targets. The highest median score was observed for *p*_α_ (Med = 0.139, IQR = 0.190), whereas the lowest was for *M* (Med = 0.034, IQR = 0.037). Age and Group appeared as predictors in two models each: Age in Sw with Be and Ac with Co, while Group in Ac with Co and As with BeCo. Group achieved the highest importance, not only compared to Age but also across all features (Med = 0.252, IQR = 0.136). Table 3 summarizes the channel-averaged feature importance scores, split by dataset and target.

**Table 3:** Feature importance scores, split for dataset and target.

** D **	** T **	**Age**	**Group**	***p*_δ_**	***p*_θ_**	***p*_α_**	***p*_β_**	***p*_γ_**	**A**	**M**	**C**
Be	Bi			0.076	0.115	0.454	0.055	0.009	0.009	0.005	0.276
Be	Sw	0.158		0.003	0.135	0.080	0.123	0.037	0.355	0.037	0.072
Co	Ac	0.088	0.388		0.035	0.048	0.084	0.116	0.039	0.030	0.173
BeCo	As		0.116	0.089	0.121	0.198	0.140	0.073	0.069	0.131	0.063

D, dataset; T, target; Age, participant's age; Group, expertise level; *p*_*B*_, normalized spectral power in band *B* = {δ, θ, α, β, γ}; *A*, activity; *M*, mobility; *C*, complexity.

**Figure 3:**
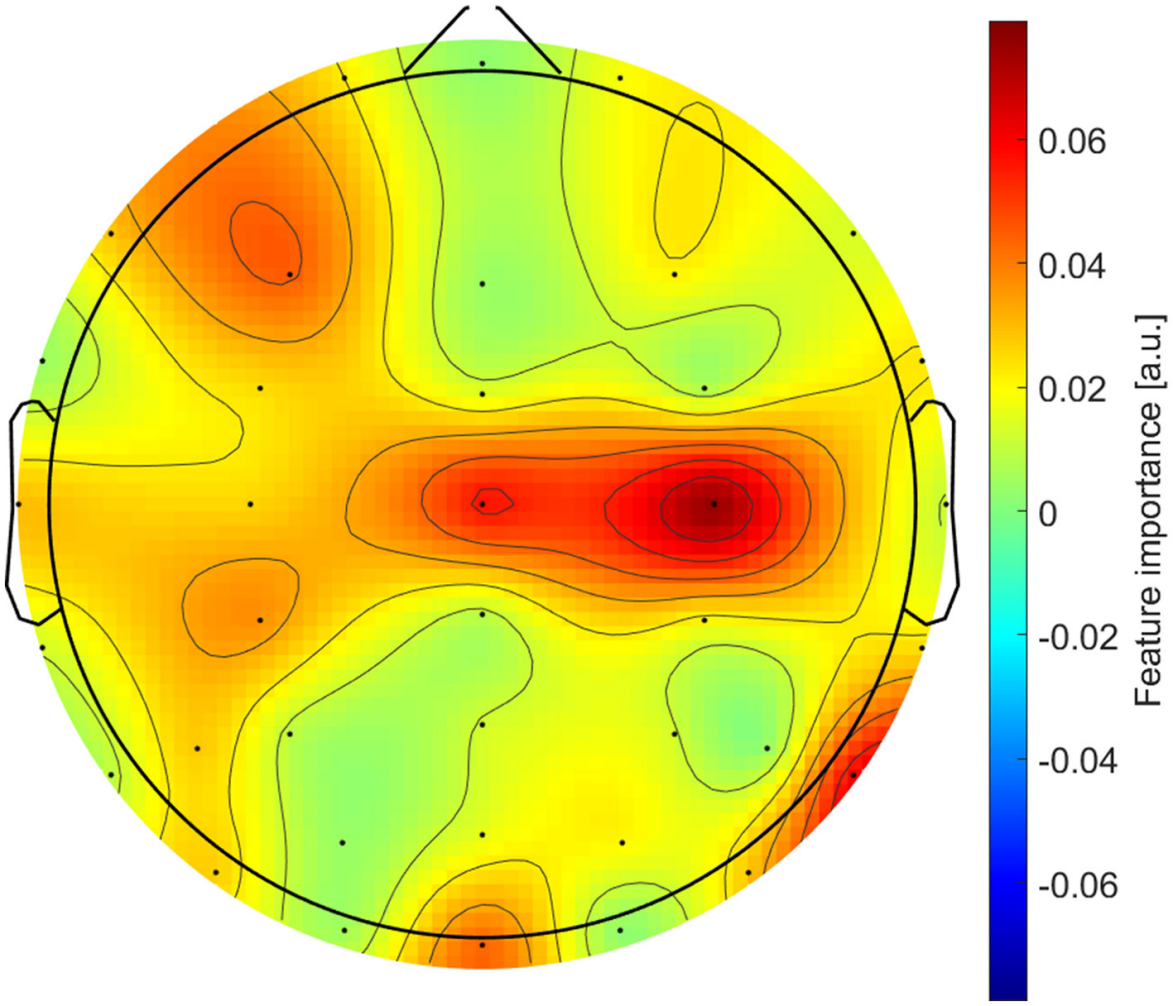
Topographic distribution of the feature importance scores.

The topographic plot in Figure 3 qualitatively identified central, occipital, parietal, and frontal regions as most important for the overall prediction.

## 5 Discussion

In this study, we trained Least-Squares Boosted Trees (LSBoost) with spectral and temporal EEG features to predict sensory attributes—bitterness (Bi), sweetness (Sw), acidity (Ac), and astringency (As)—of Coffee (Co) and basic solutions (Be). The best configuration of hyperparameters and data normalization was obtained through a Bayesian optimisation approach, following a Leave-One-Subject-Out (LOSO) scheme. The LSBoost's performances were compared with mean (Me) and lasso (LR) regressors through Cohen's *d* coefficients, and the feature importance for type and channel was assessed from the trees' coefficients.

The significant models achieved high performances, with an average anti-log MAE of about 7% of the scale range and a medium-to-large (*d*_*Me, LR*_>0.5) (Cohen, 1992) robustness against the benchmarks. The lowest MAE of Ac is in line with previous studies that identified sour as the best predictable flavor, with the highest *R*^2^ coefficient in Zhao et al. (2022) and the second (after salty) highest accuracy in Li et al. (2025). Compared to other dimensions, Sw has already shown poor performances (De et al., 2023) and low feature discriminability (Xia et al., 2024), supporting the obtained highest MAE and lowest *d* coefficients. Finally, the robustness of Bi against benchmarks may reflect the well-known evolutionary adaptation in vertebrates toward heightened bitter taste sensitivity for early toxin detection and avoidance (Wooding et al., 2021).

The feature importance of *p*_α_, *p*_β_, and *p*_γ_ is in line with past studies that effectively trained ML models using spectral powers in α, β, and γ bands (De et al., 2023; Vo et al., 2023). The role of *p*_δ_, and *p*_θ_ as key predictors is supported by previous research studies that identified differences in δ and θ bands during flavor evaluation (Wallroth et al., 2018; Yang et al., 2023). Overall, the involvement of EEG features from specific central, parietal, and frontal regions has already been observed in predictive (Li et al., 2025) and experimental (Lejap et al., 2024) studies. The contribution of temporal parameters, represented by *C, M* and *A*, matches the good performance of past deep-learning models (e.g., CNNs and RNNs) trained with the raw EEG signal (De et al., 2023; Xia et al., 2024). Finally, the significance of Group and Age could be related to the previously reported influence of expertise (Croijmans and Majid, 2016) and aging (Mojet, 2003) on sensory evaluations. However, the reasons why they impacted only in two models require further investigation.

This study acknowledges some limitations. First, despite being in line with DSA studies that typically involve 5–15 experts (Gacula and Rutenbeck, 2006), the sample size must still be considered limited. Increasing it in both magnitude and heterogeneity (e.g., adding non-expert tasters and accounting for their coffee-consumption frequency) would potentially improve not only the performance but also the generalisability of the models. An increase in dataset size would also yield less noisy results, which is particularly relevant in chemo-sensory studies. In fact, despite the use of advanced denoising techniques and the selection of short epochs with minimal muscular artifacts, the data quality in the present study should still be regarded as suboptimal. Second, the experiment has not accounted for confounders given by the fixed Be-Co order, the temperature difference between the Be and Co samples, as well as potential visual cues (e.g., the colors of the liquids), potentially biasing the sensory analyses (Delwiche, 2023). Future confirmatory studies should be, thus, based on a fully-randomized and truly-blind design. Third, our models were trained and validated in a single session per subject. Although being standard practice in multivariate-pattern-analysis with brain-imaging data (Taxali et al., 2021), it prevented us from quantifying the test-retest reliability of the models. Future works should acquire longitudinal recordings—at least a second session separated by days or weeks—to determine the stability of features and models over time.

Nevertheless, our exploratory study endorses the use of regression techniques based on EEG data in flavor assessment, as an alternative to self-report sensory evaluations.

## Data Availability

The raw data supporting the conclusions of this article will be made available by the authors, without undue reservation.
